# Grade I, II and III Follicular Lymphomas Express Ig V_H_ Genes with Different Patterns of Somatic Mutation

**DOI:** 10.1007/s12253-020-00843-x

**Published:** 2020-07-23

**Authors:** Balázs Csernus, Botond Timár, Zsolt Fülöp, András Matolcsy

**Affiliations:** 1 1st Department of Pathology and Experimental Cancer Research, Faculty of Medicine, Semmelweis University, Üllői út 26, Budapest, 1085 Hungary; 2 Department of Laboratory Medicine, Karolinska Institute, Solna, Sweden

**Keywords:** Follicular lymphoma|, Immunoglobulin gene, Somatic mutation, Clonal evolution

## Abstract

Follicular lymphoma (FL) is an indolent, B-cell, non-Hodgkin’s lymphoma with varying cytological appearance and clinical behavior. The genetic hallmark of FL is the t(14;18) translocation, and as a germinal center derived entity it is also characterized by somatic hypermutation of the immunoglobulin heavy chain (IgH) gene. In an attempt to correlate this molecular signature with the cytological grading of FL, we have analyzed the IgH variable (IgV_H_), regions in all cytological grades of FL. Four FL cases showing t(14;18) translocation were classified into grade I-III categories according to the current WHO guidelines. The IgV_H_ gene segments were PCR-amplified, sequenced, and compared to their respective germline IgV_H_ sequences. The neoplastic cells of grade I and II FLs revealed clonally related, but highly divergent IgV_H_ gene sequences indicating the ongoing nature of somatic hypermutation. Grade III FL also showed extensive presence of somatic hypermutation, but these mutations were not associated with intraclonal divergence. Thus, these results suggest that grade I-II and grade III FL may represent different biological entities. The presence of ongoing somatic hypermutation of IgV_H_ sequences in grade I and II FLs is compatible with direct follicular origin of these tumor cells, contrasting the homogenous, stable clones of grade III FL resembling a post-follicular stage of B-cell development. Our findings demonstrate that contrary to the three tiered cytological grading, molecular features of IgH genes classify FL into two distinct subcategories. These studies also suggest that with progression FL gains post-follicular–like molecular features and becomes independent of the germinal center microenvironment.

**Electronic supplementary material:**

The online version of this article (10.1007/s12253-020-00843-x) contains supplementary material, which is available to authorized users.

## Introduction

**Fig. 1.**
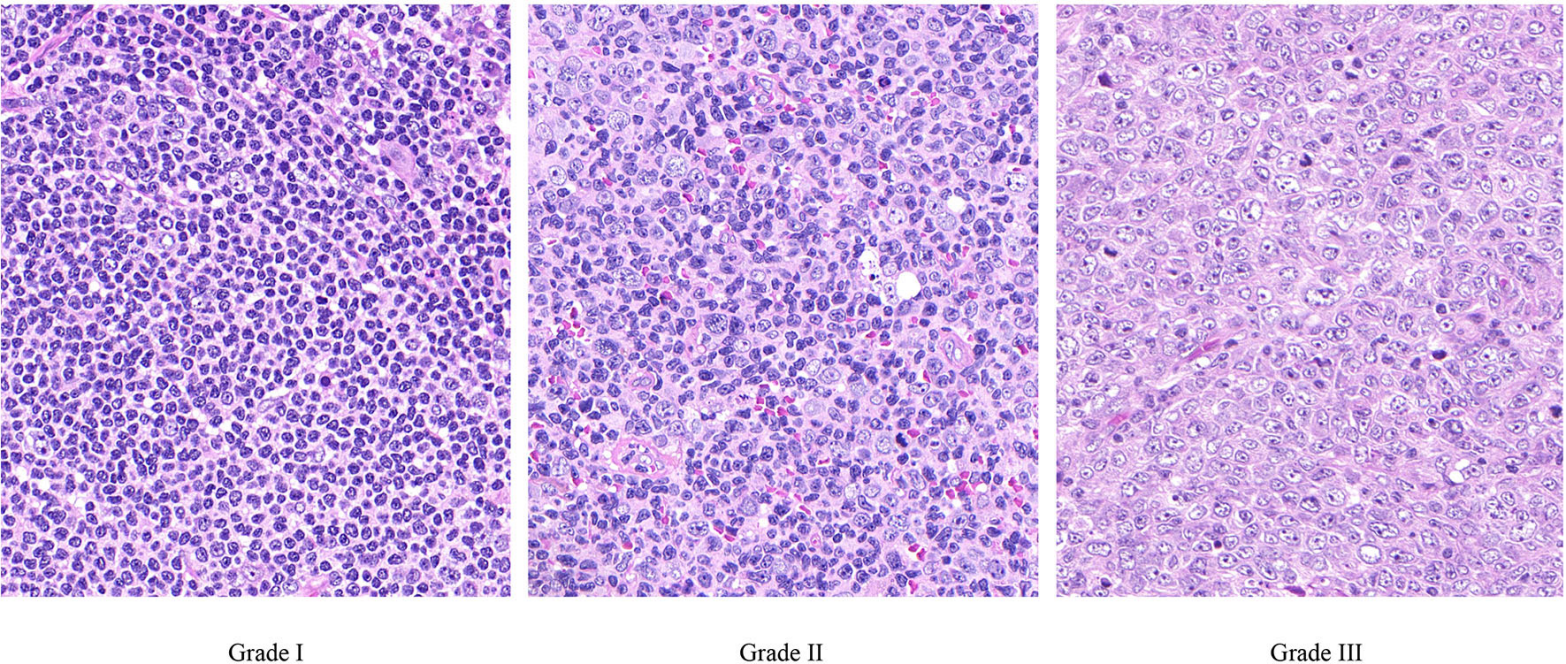
Histological appearance of the various grades of follicular lymphoma. Grade I: less than 5 centroblasts /HPF; grade II: 5–15 centroblasts /HPF; and grade III: more than 15 centroblasts/HPF. HE staining, 40x magnification (objective). Each image represents approximately 1 HPF

Follicular lymphoma (FL) is a predominantly indolent, germinal center derived, B-cell non-Hodgkin’s lymphoma with an overwhelming majority of cases carrying the characteristic t(14;18) translocation. Morphologically, FLs are composed of a mixture of centrocytes (small cleaved cells) and centroblasts (large non-cleaved cells) which grow in a highly organized nodular/follicular pattern [1–3]. Based on the proportion of centroblasts within the neoplastic follicles, FLs can be classified into grade I (less than 5 centroblasts /HPF), grade II (5-15 centroblasts /HPF and grade III (more than 15 centroblasts/HPF) categories (Fig. 1) [3, 4]. This latter category can be subdivided into grade IIIA (centrocytes are still present) and grade IIIB (centrocytes are completely absent). The histological classification or grading system of FL has been shown to correlate with the clinical prognosis suggesting that FL consist of lymphomas with different biological behavior [5–9].

B-cell lymphomas are generally considered to originate from B-lymphocytes “frozen” at certain points of their physiological differentiation [10, 11]. Molecular analysis of Ig genes in B-cell malignancies provide valuable information about stage of B-cell development at which clonal expansion had occurred. In case of follicular lymphomas, the cell of origin is considered to be the B-cells arrested at the germinal center stage of differentiation [12, 13]. This concept is supported by the findings that the immunoglobulin (Ig) genes of FL B-cells are exposed to the hypermutation machinery characterizing the physiological GC reaction. In addition, the type and distribution of these somatic IgH mutations indicate the contribution of antigen selection and reflect the clonal evolution of the neoplastic cells [14, 15].

In this study, we have analyzed Ig V_H_-D-J_H_ gene nucleotide sequences expressed by a spectrum of grade I-III FLs in order to determine whether the different grades of FL - associated with distinct clinical and biological features - show any difference in the IgH mutation patterns with respect to distribution of somatic hypermutations, and evidence for clonal evolution or antigen selection. Our results demonstrate intraclonal sequence heterogeneity caused by ongoing somatic mutation in tumor cells of grade I and II, but not in grade III FLs. These result suggest that grade I-II FLs may represent separate biological entities from grade III FL and that grade III follicular lymphoma is a genetically more stable form of the disease with possible loss intrinsic and/or less dependence on extrinsic (microenvironment) factors necessary for ongoing hypermutation of the IgH gene.

## Materials and Methods

### Tumor Biopsies

Lymph node biopsy samples from four patients with previously diagnosed follicular lymphoma were selected for this study based on the availability of paraffin tissue blocks and fresh frozen tissue for molecular analyses. All cases were categorized histologically and graded (grade I-III) according to the revised (2016) WHO classification of hematopoietic and lymphoid disease [3].

The immunophenotype of lymphoma cells was determined by the three-step avidin-biotin immunoperoxidase method using mouse anti-human monoclonal antibodies against: CD20, BCL6, BCL2, Ki67 (DAKO, Glostrup, Denmark), and CD10 (Novocastra Laboratories, Newcastle, UK) antigens. Follicular dendritic cells of the germinal centers were highlighted CD21 (Novocastra) antibodies.

The cytologic grades of the cases were grade I in case 87-784, grade II in case 94-567 and grade III in cases 91-1307 and 93-2183. The neoplastic centrocyte/centroblast population expressed CD20, CD10, BCL6 and BCL2 in all cases and demonstrated 100% follicular organization. Confirmation of the presence of the t(14,18) translocation was performed by FISH analysis using the commercially available LSI IgH/BCL2 dual-colour, dual-fusion translocation probe set (14q32, 18q21) (from Vysis, IL, USA) on at least 200 interphase nuclei.

### Isolation of Genomic DNA and RNA and First-Strand cDNA Synthesis

Genomic DNA and total RNA were extracted from frozen tissue sections using the salting-out technique and RNeasy mini kits (Qiagen), respectively. Five μg of total RNA was reverse transcribed into cDNA using M-MLV reverse transcriptase (Invitrogen, Carlsbad, CA), in conjunction with oligo-dT primers according to the manufacturer’s instructions.

### PCR Amplification, Cloning and Sequencing of the Expressed Ig V_H_-D-J_H_ Genes

cDNAs generated from RNA from each of the 4 FL cases were amplified by PCR using each of the six Ig V_H_ gene family leader-sequence specific sense primers together with the consensus J_H_ antisense primer in independent reactions as described previously [16]. PCR products were cloned in the pCR™ II vector using the TA Cloning Kit (Invitrogen, Carlsbad, CA), following the manufacturer’s instructions. DNA sequencing was performed directly from a small-scale plasmid preparation using the Sequenase version 2.0 (Applied Biosystems, Foster City, CA) system according to the manufacturer’s instructions. DNA sequences were analyzed using the MacVector version 4.5 (Eastman Kodak Co., New Haven, CT) software and the NCBI GeneBank data base.

### PCR Amplification, Cloning and Sequencing of the Genomic Ig V_H_ Genes

Genomic DNA originating from tumor samples of cases 87-784, 94-567, 91-1307 and 93-2181 were amplified to analyze the sequences of IGHV5-51, IGHV3-48, IGHV3-30 and IGHV4-39 germline genes, respectively. The appropriate sequences to be amplified in each case were obtained from sequencing results of expressed Ig V_H_-D-J_H_ genes described above. The germline IGHV5-51 gene DNA sequence was amplified using the g-IGHV5-51 sense primer [5′-TTTACCAGCTACTGGATCGGC-3′] specific for the complementary determining region (CDR) 1 sequence of IGHV5-51 gene [17] in conjunction with the antisense VH5 HEPT primer [5′-GGAATTCGCTGGTTTCTCTCACTGTG-3′] specific for the V_H_5 gene family heptamer recombination signal sequence [18]. The IGHV3-48 germline gene was amplified using the g-IGHV3-48 sense primer [5′-ACCTTCAGTAGCTATTGCATGAAC-3′] specific for the CDR1 sequence of the IGHV3-48 gene [18] in conjunction with the antisense VH3 HEPT primer [5′-GGAATTC(AC)TG(AG)C(CT)TCCCCTC(AG)CT(CG)-3′] specific for the V_H_3 gene family heptamer recombination signal sequence [18]. The IGHV3-30 germline gene was amplified using the g-IGHV3-30 sense primer [5′-TTCAGTAGCTATGCTATGCAC-3′] specific for the CDR1 sequence of the IGHV3-30 gene [19] in conjunction with the antisense VH3 HEPT primer [18]. The IGHV4-39 germline gene was amplified using the g-IGHV4-39 sense primer [5′-AGCAGTAGTAGTTACTACTGGGGC-3′] specific for the CDR1 sequence of the IGHV4-39 gene [20] in conjunction with the antisense VH4 HEPT primer [5′-GGAATTCACTCACCTCCCCTCACTGTG −3′] specific for the V_H_4 gene family heptamer recombination signal sequence [18]. Thirty cycles of PCR amplifications were performed. Each cycle consisted of denaturation (94 °C for 1 min.), annealing (58 °C for 1 min.), and extension (72 °C for 2 min.) step.

### Clonotype-Specific PCR Amplification of Ig V_H_-D-J_H_ Gene Segment Expressed by FL Cells of Case 94-567, 91-1307 and 93-2181

A PCR based approach was installed to verify whether unmutated progenitor clonotype of the FL cells were represented among neoplastic cells in case 94-567, 91-1307 and 93-2181. In these amplifications, the g-IGHV3-48, g-IGHV3-30 and g-IGHV4-39 sense primers specific for the unmutated CDR1 sequences of IGHV3-48, IGHV3-30 and IGHV4-39 gene segments respectively, and the consensus J_H_ antisense primer were used. Thirty cycles of amplifications were performed. Each cycle consisted of denaturation (94 °C for 1 min.), annealing (55 °C for 1 min.) and extension (72 °C for 2 min.) step. Amplified Ig V_H_-D-J_H_ gene DNAs were analyzed in 2.0% agarose gel electrophoresis containing 1 μg/ml ethidium bromide.

### Analysis of Mutations

We calculated the number of expected replacement (R) mutations in the CDRs and FRs of the IgV_H_ gene sequences using the formula R_CDR_ or R_FR_ = *n x (CDR Rf or FR Rf) x (CDR*_*rel*_ *or FR*_*rel*_*)*, where *n* is the total number of observed mutations, and *Rf* is the replacement frequency inherent to each IgV_H_ gene [21], and *CDR*_*rel*_ and *FR*_*rel*_ are the relative size of the CDRs and FRs, respectively. A binomial probability model was used to evaluate whether the excess and paucity of R mutations in CDRs and FRs, respectively were due to chance only: *p* = *{n!/[k!(n-k)!]} X q*^*k*^ *X (1-q)*^*n-k*^, where *q* = the probability that an R mutation will localize to CDRs or FRs (*q* = *CDR*_*rel*_ x *CDR Rf* or *FR*_*rel*_ x *FR Rf*), and *k* = the number of observed R mutations in the CDRs or FRs [21].

## Results

### Ig V_H_-D-J_H_ Gene Sequences Expressed by FL Cells

Using individual Ig V_H_ gene family-specific leader sense primers in conjunction with the consensus J_H_ antisense primer, the Ig V_H_-D-J_H_ segments were PCR-amplified from cDNA in six independent reactions in each case. The PCR products were cloned, and 12 independent bacterial isolates (labelled A-L) were sequenced and analyzed from each case. The nucleotide and deduced amino acid sequences of the V_H_-D-J_H_ gene segments, and those of the closest respective germline gene sequences are depicted in supplementary Figs. 1 and 2, and summarized in Table 1.

**Table 1.** Analysis of Ig V_H_-D-J_H_ genes expressed by neoplastic cells of  the four FL cases

			Ig VH Gene									
Case No.	Clone	Intraclonal Diversity	Closest Germline Gene	Nucleotide Identity %	CDR1 and CDR2			FR1, FR2 and FR3			D	JH
					R	*p*	S	R	*p*	S	Gene	Gene
87-784	A–C	yes	IGHV5-51	100.0	0 (0.00)	0.00	0	0 (0.00)	0.00	0	ND	HJ4
	D–L	yes	IGHV5-51	97.6	5 (1.74)	1.56 × 10^–2*^	3	2 (5.94)	1.17 × 10^–2*^	0	ND	HJ4
94-567	A–C	yes	IGHV3-48	93.9	9 (3.26)	1.68 × 10^–3*^	3	4 (10.28)	2.33 × 10^–3*^	2	ND	HJ4
	D	yes	IGHV3-48	93.6	10 (3.44)	5.78 × 10^–4*^	3	4 (10.85)	1.27 × 10^–3*^	2	ND	HJ4
	E	yes	IGHV3-48	93.9	9 (3.26)	1.68 × 10^–3*^	3	4 (10.28)	2.33 × 10^–3*^	2	ND	HJ4
	F	yes	IGHV3-48	93.2	10 (3.62)	9.47 × 10^–4*^	3	4 (11.42)	6.79 × 10^–4*^	3	ND	HJ4
	G	yes	IGHV3-48	92.9	14 (3.80)	1.00 × 10^–6*^	3	2 (11.99)	7.00 × 10^–6*^	2	ND	HJ4
	H	yes	IGHV3-48	92.6	15 (3.98)	1.00 × 10^–6*^	3	2 (12.56)	3.00 × 10^–6*^	2	ND	HJ4
	I	yes	IGHV3-48	92.2	15 (4.16)	1.00 × 10^–6*^	3	2 (13.13)	2.00 × 10^–6*^	3	ND	HJ4
	J	yes	IGHV3-48	92.6	14 (3.98)	3.00 × 10^–6*^	3	3 (12.56)	3.00 × 10^–5*^	2	ND	HJ4
	K	yes	IGHV3-48	91.8	14 (4.34)	1.10 × 10^–5*^	5	3 (13.70)	7.00 × 10^–6*^	2	ND	HJ4
	L	yes	IGHV3-48	91.1	15 (4.71)	6.00 × 10^–6*^	4	5 (14.84)	7.70 × 10^–5*^	2	ND	HJ4
91-1307	A–L	no	IGHV3-30	91.8	8 (4.28)	3.25 × 10^–2*^	4	9 (13.78)	2.43 × 10^–2*^	3	ND	HJ4
93-2181	A–L	no	IGHV4-39	90.2	7 (5.22)	1.12 × 10^−1^	3	14 (15.98)	1.13 × 10^−1^	4	ND	HJ6

ND, not detected; R, number of detected and (expected) replacement mutations; S, number of detected silent mutations; *p*, probability

* statistically significant (*P* < 0,05)

In case 87-784 (representing grade I FL), V_H_-D-J_H_ gene sequences derived from 12 independent isolates revealed two unique, but collinear sequences (87-784/A-C and 87-784/D-L) sharing the same V_H_, (D), and J_H_ genes and joinings. These V_H_-D-J_H_ gene sequences displayed the highest degree of identity to those of the germline IGHV5-51 and HJ4 genes [17]. The intervening sequence could not be attributed to any germline D gene. The expressed IgV_H_ genes of clones 87-784/A-C were 100% identical to those of the germline IGHV5-51 gene, but clones 87-784/D-L displayed 10 nucleotide differences when compared to germline.

The analysis of the 12 independent isolates derived from the FL cells of case 94-567 (representing grade II FL) revealed 10 unique, but collinear V_H_-D-J_H_ sequences sharing the same V_H_, (D), and J_H_ genes and joinings. These V_H_-D-J_H_ gene sequences displayed the highest degree of identity to those of the germline IGHV3-48 and HJ4 genes [18]. The intervening sequences could not be attributed to any germline D gene. The expressed V_H_ genes revealed 91.1–93.9% homology to the reported germline IGHV3-48 sequence.

All nucleic acid sequences derived from the FL cells of case 91-1307 (representing grade III FL) were found to be identical. The expressed V_H_-D-J_H_ genes displayed the highest degree of identity to those of the germline IGHV3-30 and HJ4 genes, respectively [19]. The intervening sequences could not be attributed to any germline D gene. The V_H_ gene sequence showed a 24 nucleotide difference compared to the reported germline IGHV3-30 gene.

In case 93-2181 (representing grade III FL), V_H_-D-J_H_ gene sequences derived from the tumor cells were also identical. The expressed V_H_-D-J_H_ genes displayed the highest degree of identity to those of the germline IGHV4-39 and HJ6 genes [20]. The intervening sequence could not be attributed to any germline D gene. The V_H_ gene sequence showed a 28 nucleotide difference from the reported germline IGHV4-39 gene.

**Fig. 2:**
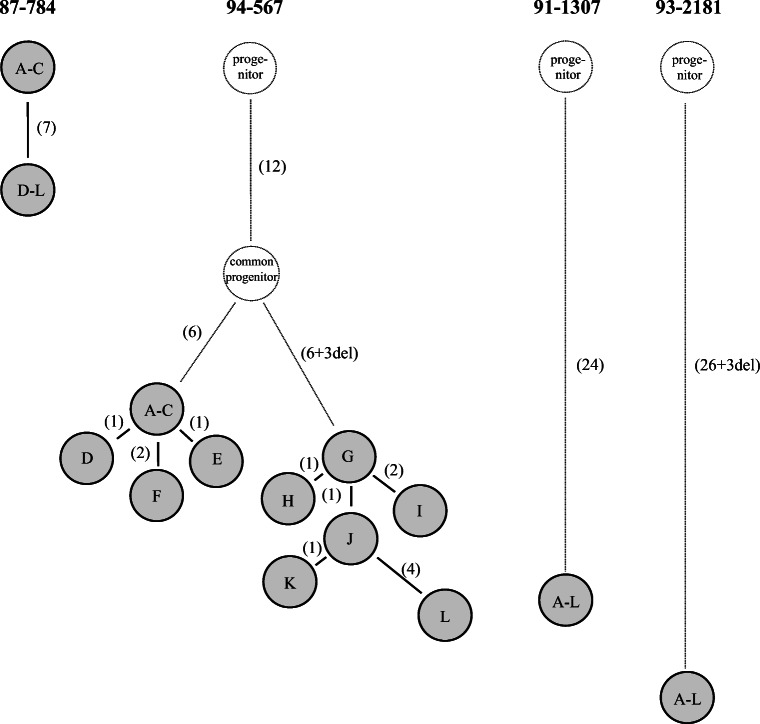
Presumed clonal evolution of the four FL cases. Genealogical trees are constructed from the pattern of shared and unique mutations of Ig V_H_-D-J_H_ genes, assuming that shared mutations represent single events. The deducted common progenitors and the intermediates are drawn as dotted circles. The number of mutations separating each branch are given in parentheses. The distances between the circles are proportional to the number of mutations

In all the four FL cases, the expressed V_H_-D-J_H_ gene sequences were aligned and the sequence of their putative FL cell progenitor was calculated. From the pattern of shared and unique mutations - assuming that shared mutations represent single events and not independent mutations - genealogical trees of the evolution of FL cells were constructed, and putative progenitor IgH gene sequences were deduced (Fig. 2).

### Identification of the Germline IGHV5-51, IGHV3-48, IGHV3-30 and IGHV4-39 in Cases 87-784, 94-567, 91-1307 and 93-2181

The IgV_H_ gene sequences expressed by the neoplastic B-cells in cases 87-784, 94-567, 91-1307 and 93-2181 were 97.6 to 100%, 91.1 to 93.9%, 91.8% and 90.2% identical to those of the reported germline IGHV5-51, IGHV3-48, IGHV3-30 and IGHV4-39 genes respectively, and supposedly represented somatically mutated forms of these V_H_ gene segments. To verify that copies of these germline genes truly existed in the genome and to rule out the confounding effects of single nucleotide polymophisms, genomic DNAs from the tumor samples of all four patients were PCR-amplified using sense primers specific for germline CDR1 sequences in conjunction with antisense primers specific for V_H_ gene family heptamer recombination signal sequences (as described in materials and methods). Thus, our PCR reactions were specifically designed to only amplify the germline configurations of the V_H_ genes, since the heptamer recombination signal sequences are lost in the rearranged IgV_H_ genes. Using these oligonucleotide primer pairs, we were able to obtain PCR products of the appropriate size in all four cases, indicating that the tumor samples contained some non-neoplastic cells. In each case, the PCR products were cloned, and plasmid DNAs originated from six independent bacterial isolates were sequenced. In case 87-784, the nucleotide sequences of the six independent isolates were 100% identical to that of the germline IGHV5-51 gene (supplementary Figs. 1 and 2). In case 94-567, two cloned plasmids contained inserts identical in sequence to that of the IGHV3-48 germline gene and four sequences identical to IGHV3–51 germline gene. In case 91-1307, the nucleotide sequences of the six independent isolates were 100% identical to that of the germline IGHV3-30 gene (supplementary Fig. 1). In case 93-2181, three cloned plasmids contained inserts identical in sequence to that of the IGHV4-4 germline gene, the remaining three clones incorporated sequences that were identical to IGHV4-39 germline gene. Thus, these findings are consistent with the hypothesis that the Ig V_H_ gene segments expressed by neoplastic cells of cases 87-784, 94-567, 91-1307 and 93-2181 are somatically mutated forms of genomic IGHV5-51, IGHV3-48, IGHV3-30 and IGHV4-39 genes.

### Search for Unmutated FL Progenitor Clonotypes in Cases 94-567, 91-1307 and 93-2181

The sequence analysis of Ig V_H_-D-J_H_ gene expressed by the tumor cells of case 87-784 showed that 87-784/D-L clones derived from 87 to 784/A-C progenitors. The 87-784/A-C clones contained rearranged and unmutated IGHV5-51 genes. Comparable analysis of the FL cells in cases 94-567, 91-1307 and 93-2181 revealed only somatically mutated clonotypes, and unmutated common progenitor cells were completely absent. To determine whether remaining unmutated progenitors are still present among the somatically mutated FL cells, or these cells were completely lost during the clonal evolution of FL, we developed clonotype specific PCR reactions identifying the unmutated and expressed IgV_H_ gene sequences. For this PCR amplification we used the germline CDR1 sequence specific sense primers in conjunction with the consensus J_H_ antisense primer as described in materials and methods. No DNA amplifications were detected using this approach in cases 94-567, 91-1307 and 93-2181 (results not shown). These data suggest that unmutated common progenitor cells were lost during the clonal evolution in all our FL cases.

### Analysis of Somatic Mutations Detected in the Expressed V_H_ Genes

The number of expected replacement (R) mutations and the probability that these R mutations in the CDR or FR regions arose by chance were calculated for all four FL cases (Table 1). In cases 87-784, 94-567 and 91-1307 the number of R mutations in the CDR1 and CDR2 regions were significantly higher than expected (*p* < 0,05), while in the FR1, FR2 and FR3 regions they were lower than expected. This indicates that the likelihood that these R mutations in the CDRs and FRs occurred randomly is low. Thus, the higher than expected rate of R mutations in the CDRs is consistent with antigen selection in clones of 87-784/D-L, 94-567/A-L and 91-1307, providing genetic evidence that these IgV_H_ gene segments were under positive selective pressure to mutate their CDRs, but negative pressure to mutate their FRs. In the case number 93-2181 the number of R mutations in the CDR and FR were similar to that expected by chance alone, suggesting the lack positive selective pressure for R mutations in this Ig V_H_ gene.

## Discussion

FL can be classified according to the WHO into grade I-III categories based on histological features that correlate with clinical outcome. Several large studies have identified numerous recurrent mutations affecting the B-cell receptor signaling and differentiation, cell cycle regulation, derangements of epigenetic modification and immune evasion that can be linked to clinical progression of the disease [22–25], however the mechanism of clonal evolution is still a matter of debate [26, 27]. The IgV_H_ somatic hypermutation based spatial evolution of follicular lymphoma have been demonstrated previously [28, 29]. In our study we have analyzed the sequence of Ig V_H_-D-J_H_ genes in different histological grades of FL in an attempt to reveal differing molecular signatures correlating with the histological appearance and biological behavior. We have found that grade I-II FLs, consisting predominantly centrocytes and grade III FLs, containing numerous centroblasts, express Ig V_H_-D-J_H_ gene sequences with different patterns of somatic mutation. Ongoing somatic mutation and intraclonal diversity were detected in the low cytological grades (grade I and II) of FL indicating that the tumor cells are still under the influence of the mutation machinery, possibly generated through interactions with their environment in the GC or GC analogous milieu, during the course of neoplastic transformation. In contrast, grade III FLs expressed mutated, but uniform V_H_-D-J_H_ gene sequences, suggesting that the previously active mutational mechanism has already been terminated, and the lymphoma cells have become less dependent on GC-like environmental stimulation for survival.

The process of active somatic hypermutation of Ig genes, which occurs in germinal centers during normal B-cell differentiation, has been suggested to be a common feature of FLs [28, 30–32]. Our results partially support these observations, and demonstrate that the neoplastic cells of grade I and II FLs are subjected to extensive ongoing somatic hypermutation of the Ig V_H_ genes resulting in significant intraclonal heterogeneity. In our grade I FL case, the V_H_-D-J_H_ gene sequences segregated into two clonally related clusters. The V_H_ gene sequence of the first cluster was 100% homologous to germline gene, while the second cluster displayed a 10 nucleic acid difference. In the grade II FL case, 10 clonally related, but intraclonally divergent sequences were detected. The V_H_ gene sequence of the neoplastic clones showed 91.1-93.9% sequence homology to the germline gene. The shared and non-shared nucleotide alterations detected in the different neoplastic clones suggest a stepwise accumulation of mutations paralleling the evolution of the FL clones. The pattern of sequence polymorphism allowed the construction of genealogical trees, where the individual subclones differed from the common progenitor by progressively accumulated nucleotide changes. Thus, these results confirm previous findings that clonal expansion of low-grade (grade I and II) FL is still associated with ongoing somatic hypermutation of the IgV_H_ gene.

The intensely mutated, but identical V_H_-D-J_H_ gene sequences detected in grade III FLs are strikingly different from the intraclonally divergent subclones of grade I and II FLs. The V_H_ gene sequences expressed by grade III FL revealed a 24 nucleic acid difference in one case, and a 26 base pair difference plus three nucleic acid deletions in the other case compared to the germline. These findings suggest that the development of grade III FLs originate from a late neoplastic clone with numerous previously acquired V_H_ gene mutations, rather than from the outgrowth of an early more germline-like clone. More V_H_ gene mutations translate to longer exposure to the highly mutagenic GC environment, which possibly also allows for stepwise accumulation of transforming mutations of the key regulatory genes through aberrant somatic hypermutation. The lack of evidence for ongoing mutations in the higher-grade cases suggest that these malignant cells have become independent of the environmental influence of the germinal center, and the neoplastic clone is no longer exposed to the mutation machinery. Interestingly, these features are usually characteristic for lymphomas originating from the post-follicular stage of B cell development.

The higher number of Ig V_H_ gene mutations in grade III FLs enriched for centroblast like large cells compared to lower grade FL dominated by centrocytes, was unexpected. The V_H_ gene sequence analysis of microdissected centroblasts and centrocytes from reactive germinal centers showed contrasting features [33]. The large centroblasts of the dark zone of a reactive GC express germline-encoded V regions and small centrocytes of the light zone of GC express mutated V regions. These data suggest that the neoplastic centroblasts and centrocytes of FL are not analogous to centroblasts and centrocytes of normal CG at the genetic level.

The study presented here demonstrates the different patterns of somatic mutation in the different grades of FLs. However, the question of tumor clone development is still unanswered. There are two putative models that may possibly explain the different patterns of somatic mutation detected in different grades FL. In the first model, FL is a heterogeneous disorder originating from different committed precursor cells at various stages of B-cell development. Based on this hypothesis, grade I and II FLs are transformants of germinal center B cells, while grade III FLs originate from a post-follicular (memory) B cell. This hypothesis is also supported by the finding that we were unable to confirm the presence of a common progenitor clonotype in grade III FL by clonotype specific PCR amplification. A recent demonstration that in situ follicular lymphoma (ISFL), overt FL and transformed FL often lack a clonal relationship, also supports the theory of multiple committed precursors that may undergo unique changes creating different malignant clones [22, 34]. Gene expression profiling has also revealed significantly different expression signatures between low-grade (I and II) and high-grade (IIIA and IIIB) FL suggesting that grade I-II and grade III FL may represent different biological entities. [35].

Alternatively, all FLs may originate from a single committed progenitor driven by the presence of t(14,18) translocation, and following malignant transformation, ongoing (aberrant) somatic hypermutation gives rise to sequential appearance of different grades of FLs. According to this model, the grades of FLs represent different time points of the tumor clone evolution. This hypothesis is supported by the finding that in our low-grade FLs unmutated progenitor clonotypes gave rise to a mutated grade I FL subclone, and that according to our results, the number of mutations show progressive increase with grade. Evidence for clonal relatedness following transformation of FL into a high-grade lymphoma (DLBCL) has also been documented previously [26, 36, 37]. In this scenario, the early phases of progression could be associated with ongoing somatic mutation that may freeze-up during the process of high-grade transformation along with loss of the environmental interactions or possible functional loss the mutation machinery.

## Electronic supplementary material

ESM 1 (DOC 38 kb)

ESM 2 (DOC 26 kb)
