# Supplementary material for: Grade I, II and III Follicular Lymphomas Express Ig VH Genes with Different Patterns of Somatic Mutation
Source: Pathol Oncol Res. 2020 Jul 23;26(4):2765–72. doi: 10.1007/s12253-020-00843-x (PMC7471144; doi:10.1007/s12253-020-00843-x)
Supplement: Supplementary file 1 — (DOC 38 kb) [file 12253_2020_843_MOESM1_ESM.doc]

**___________CDR1____________**

**1 10 20 30 35A 35B 40**

**IGHV5-51 GAG GTG CAG CTG GTG CAG TCT GGA GCA GAG GTG AAA AAG CCC GGG GAG TCT CTG AAG ATC TCC TGT AAG GGT TCT GGA TAC AGC TTT ACC AGC TAC TGG ATC GGC --- --- TGG GTG CGC CAG ATG CCC GGG AAA GGC CTG GAG TGG ATG GGG**

**87-784(GL) g-HV5-51 ... ... ... ... ... ... ... ... --- --- ... ... ... ... ... ... ... ... ... ... ... ... ... ...**

**87-784/A-C ... ... ... ... ... ... ... ... ... ... ... ... ... ... ... ... ... ... ... ... ... ... ... ... ... ... ... ... ... ... ... ... ... ... ... --- --- ... ... ... ... ... ... ... ... ... ... ... ... ... ...**

**87-784/D-L ... ... ... ... ... ... ... ... ... ... ... ... ... ... ... ... ... ... ... ... ... ... ... ... ... ... ... ... ... ... .A. ... ... ... .C. --- --- ... ... ... ... ... ... ... ... ... ... ... ... ... ...**

**IGHV3-48 GAG GTG CAG CTG GTG GAG TCT GGG GGA GGC TTG GTA CAG CCT GGG GGG TCC CTG AGA CTC TCC TGT GCA GCC TCT GGA TTC ACC TTC AGT AGC TAT AGC ATG AAC --- --- TGG GTC CGC CAG GCT CCA GGG AAG GGG CTG GAG TGG GTT TCA**

**94-567(GL) g-HV3-48 ... ... ... ... ... ... ... ... --- --- ... ... ... ... ... ... ... ... ... ... ... ... ... ...**

**94-567/A-C ... ... ... ... ... ... ... ... ... ... ... ... ... ... ... ... ... ... ... ... ... ... ... ... ... ... ... ... ... ... GA. .T. .CT C.. ... --- --- ... ... ... ... ... ... ... ... ... ... ... ... A.. ...**

**94-567/D ... ... ... ... ... ... ... ... ... ... ... ... ... ... ... ... ... ... ... ... ... ... ... ... ... ... ... ... ... ... GA. .T. .CT C.. ... --- --- ... ... ... ... ... ... ... ... ... ... ... ... A.. ...**

**94-567/E ... ... ... ... ... ... ... ... ... ... ... ... ... ... ... ... ... ... ... ... ... ... ... ... ... ... ... ... ... ... GA. .T. .CT C.. ... --- --- ... ... ... ... ... ... ... ... ... ... ... ... A.. ...**

**94-567/F ... ... ... ... ... ... ... ... ... ... ... ... ... ... ... ... ... ... ... ... ... ... ... ... ... ... ... ... ... ... GA. .T. .CT C.. ... --- --- ... ... ... ... ... ... ... ... ... ... ... ... A.. ...**

**94-567/G ... ... ... ... ... ... ... ... ... ... ... ... ... ... ... ... ... ... ... ... ... ... ... ... ... ... ... ... ... ... GA. .T. TCT C.. ..T --- --- ... ... ... ... ... ... ... ... ... ... ... ... A.. ...**

**94-567/H ... ... ... ... ... ... ... ... ... ... ... ... ... ... ... ... ... ... ... ... ... ... ... ... ... ... ... ... ... ... GA. .T. TCT C.. ..T --- --- ... ... ... ... ... ... ... ... ... ... ... ... A.. ...**

**94-567/I ... ... ... ... ... ... ... ... ... ... ... ... ... ... ... ... ... ... ... ... ... ... ... ... ... ... ... ... ... ... GA. .T. TCT C.. ..T --- --- ... ... ... ... ... ... ... ... ... ... ... ... A.. ..G**

**94-567/J ... ... ... ... ... ... ... ... ... ... ... ... ... ... ... ... ... ... ... ... ... ... ... ... ... ... ... .T. ... ... GA. .T. TCT C.. ..T --- --- ... ... ... ... ... ... ... ... ... ... ... ... A.. ...**

**94-567/K ... ... ... ... ... ... ... ... ... ... ... ... ... ... ... ... ... ... ... ... ... ... ... ... ... ... ... .T. ... ... GA. .T. TCT C.. ..T --- --- ... ... ... ... ... ... ... ... ... ... ... ... A.. ...**

**94-567/L ... ... ... ... ... ... ... ... ... ... ... ... ... ... ... ... ... ... ... ... ... ... ... ... ... ... ... .T. ... ... GA. .TC TCT C.. ..T --- --- ..C ... ... ... ... ... ... ... ... ... ... ... A.. ...**

**IGHV3-30 CAG GTG CAG CTG GTG GAG TCT GGG GGA GGC GTG GTC CAG CCT GGG AGG TCC CTG AGA CTC TCC TGT GCA GCC TCT GGA TTC ACC TTC AGT AGC TAT GCT ATG CAC --- --- TGG GTC CGC CAG GCT CCA GGC AAG GGG CTA GAG TGG GTG GCA**

**91-1307(GL) g-HV3-30 ... ... ... ... ... ... ... --- --- ... ... ... ... ... ... ... ... ... ... ... ... ... ...**

**91-1307/A-L ... ... ... ... ... ... ... ... ... ... ... ... ... ... ... ... ... ... ... ... ... ... ... ... ... ... ... ... ... ... C.. ... A.. ... TTT --- --- ... ... ... ... ... ... ... ... ... ..G ... ... ... ...**

**IGHV4-39 CAG CTG CAG CTG CAG GAG TCG GGC CCA GGA CTG GTG AAG CCT TCG GAG ACC CTG TCC CTC ACC TGC ACT GTC TCT GGT GGC TCC ATC AGC AGT AGT AGT TAC TAC TGG GGC TGG ATC CGC CAG CCC CCA GGG AAG GGG CTG GAG TGG ATT GGG**

**93-2181(GL) g-HV4-39 ... ... ... ... ... ... ... ... ... ... ... ... ... ... ... ... ... ... ... ... ... ...**

**93-2181/A-L ... ... ... ... ... ... ... ... ... ... ... ... ... ... ... ... ... ... ... ... ... ... ... ... ... ... ... ... ..A ... ... .A. .T. C.. ... ... ... ... ... ... ... --- ... ... ... ..A ... ... ... ... ...**

**_______________________________CDR2________________________________**

**50 52 52A 60 70 80 82 82A 82B 82C 83 90**

**IGHV5-51 ATC ATC TAT CCT GGT GAC TCT GAT ACC AGA TAC AGC CCG TCC TTC CAA GGC CAG GTC ACC ATC TCA GCC GAC AAG TCC ATC AGC ACC GCC TAC CTG CAG TGG AGC AGC CTG AAG GCC TCG GAC ACC GCC ATG TAT TAC TGT GCG AGA**

**87-784(GL) ... ... ... ... ... ... ... ... ... ... ... ... ... ... ... ... ... ... ... ... ... ... ... ... ... ... ... ... ... ... ... ... ... ... ... ... ... ... ... ... ... ... ... ... ... ... ... ... ...**

**87-784/A-C ... ... ... ... ... ... ... ... ... ... ... ... ... ... ... ... ... ... ... ... ... ... ... ... ... ... ... ... ... ... ... ... ... ... ... ... ... ... ... ... ... ... ... ... ... ... ... ... ...**

**87-784/D-L ..G ... ... ... CA. ... ... ... ... ... ... ... ... ... ... ... ... ... ... ... ... ... ... ... ... ... C.. .C. ... ... ... ... ... ... ... ... ... ... ... ... ... ... ... ... ... ... ... ... ...**

**IGHV3-48 TAC ATT AGT AGT AGT AGT AGT ACC ATA TAC TAC GCA GAC TCT GTG AAG GGC CGA TTC ACC ATC TCC AGA GAC AAT GCC AAG AAC TCA CTG TAT CTG CAA ATG AAC AGC CTG AGA GCC GAG GAC ACG GCT GTG TAT TAC TGT GCG AGA**

**94-567 (GL) ... ... ... ... ... ... ... ... ... ... ... ... ... ... ... ... ... ... ... ... ... ... ... ... ... ... ... ... ... ... ... ... ... ... ... ... ... ... ... ... ... ... ... ... ... ... ... ... ...**

**94-567/A-C A.T ... ... ... ... ... .C. ... .C. A.. ... ..G ... ... ... ... ... ... ... ... G.. ... ... ... ... .T. ... ... ... T.. ... ... ... ... ... ... ... ... ... ... ... ... ... ..C ... ..T ... ... ...**

**94-567/D A.T ... ... ... .C. ... .C. ... .C. A.. ... ..G ... ... ... ... ... ... ... ... G.. ... ... ... ... .T. ... ... ... T.. ... ... ... ... ... ... ... ... ... ... ... ... ... ..C ... ..T ... ... ...**

**94-567/E A.T ... ... ... ... ... .C. ... .C. A.. ... ..G ... ... ... ... ... ... ... ... G.. ... ... ... ... .T. ... ... ... T.. ... ... ... ... ... ... ... ... ... ... ... ... ... ..C ... ..T ... ... ...**

**94-567/F AGT ... ... ... ... ... .C. ... .C. A.. ... ..G ... ... ... ... ... ... ... ... G.. ... ... ... ... .T. ... ... ... T.. ... ... ... ... ... ... ... ... ... ... ... ... ... ..C ..C ..T ... ... ...**

**94-567/G A.T ... ... --- ... ... .A. .T. .C. G.. ... ... ... ... ... ... ... ... ... ... ... ... ... ... ... ... ... ... ... ... ... ... ... ... ... ... ... ... .T. ... ... ... ... ..C ... ..T ... ... ...**

**94-567/H A.T ... .C. --- ... ... .A. .T. .C. G.. ... ... ... ... ... ... ... ... ... ... ... ... ... ... ... ... ... ... ... ... ... ... ... ... ... ... ... ... .T. ... ... ... ... ..C ... ..T ... ... ...**

**94-567/I A.T ... ... --- ... ... .A. .T. .C. G.. ... .T. ... ... ... ... ... ... ... ... ... ... ... ... ... ... ... ... ... ... ... ... ... ... ... ... ... ... .T. ... ... ... ... ..C ... ..T ... ... ...**

**94-567/J A.T ... ... --- ... ... .A. .T. .C. G.. ... ... ... ... ... ... ... ... ... ... ... ... ... ... ... ... ... ... ... ... ... ... ... ... ... ... ... ... .T. ... ... ... ... ..C ... ..T ... ... ...**

**94-567/K A.T ..C ... --- ... ... .A. .T. .C. G.. ... ... ... ..C ... ... ... ... ... ... ... ... ... ... ... ... ... ... ... ... ... ... ... ... ... ... ... ... .T. ... ... ... ... ..C ... ..T ... ... ...**

**94-567/L A.T ..G ... --- ... ... .A. .T. .C. G.. ... ... ... ... ... ... ... ... ... ... ... ... ... ... ... .G. ... ... ... ... ... ... ... ... ... ... ... ... .T. ... ... ... ... ..C ... ..T ... ... ...**

**IGHV3-30 GTT ATA TCA TAT GAT GGA AGT AAT AAA TAC TAC GCA GAC TCC GTG AAG GGC CGA TTC ACC ATC TCC AGA GAC AAT TCC AAG AAC ACG CTG TAT CTG CAA ATG AAC AGC CTG AGA GCT GAG GAC ACG GCT GTG TAT TAC TGT GCG AGA**

**91-1307(GL) ... ... ... ... ... ... ... ... ... ... ... ... ... ... ... ... ... ... ... ... ... ... ... ... ... ... ... ... ... ... ... ... ... ... ... ... ... ... ... ... ... ... ... ... ... ... ... ... ...**

**91-1307/A-L C.. ..T ..G ... ... ... .A. ... G.. C.T ... ... ... ... ... ... ... ... .G. ... ..T ... ... ... ... ... .G. G.. ... ... ... ... ... T.. ... ... ... ... CT. A.C ... ... ... ..A ... ... ... ... .C.**

**IGHV4-39 AGT ATC TAT --- TAT AGT GGG AGC ACC TAC TAC AAC CCG TCC CTC AAG AGT CGA GTC ACC ATA TCC GTA GAC ACG TCC AAG AAC CAG TTC TCC CTG AAG CTG AGC TCT GTG ACC GCC GCA GAC ACG GCT GTG TAT TAC TGT GCG AGA**

**93-2181(GL) ... ... ... --- ... ... ... ... ... ... ... ... ... ... ... ... ... ... ... ... ... ... ... ... ... ... ... ... ... ... ... ... ... ... ... ... ... ... ... ... ... ... ... ... ... ... ... ... ...**

**93-2181/A-L .C. .AA ... --- ... ... ..T .TT ... ... ... ... ... ... ... .T. ... ... ... ... G.. ... .CT ... ... ... ..C ..T .GA ... ... ... .GC ... .C. ... T.. ... ... ... ... ... ... ..A ... .T. ... ... ..T**

**___________________________CDR3________________________________**

**100 00A 00B 00C 00D 00E 00F 00G 110**

**87-784/A-C CTC GAA AAC TAT GGG CAT CAT --- --- --- --- --- --- --- AAC TAC TGG GGC CAG GGA ACG CTG GTC ACC GTC TCC TCA**

**87-784/D-L ... ... ... ... ... ... ... --- --- --- --- --- --- --- ... ... ... ... ... ... ... ... ... ... ... ... ...**

**94-567/A-C AAC TCG TCC GCT --- --- --- --- --- --- --- --- --- --- GAC TAC TGG GGC CAG GGA ACG CTG GTC ACC GTC TCC TCA**

**94-567/D ... ... ... ... --- --- --- --- --- --- --- --- --- --- ... ... ... ... ... ... ... ... ... ... ... ... ...**

**94-567/E ... ... ... ... --- --- --- --- --- --- --- --- --- --- ... ... ... .A. ... ... ... ... ... ... ... ... ...**

**94-567/F ... ... ... ... --- --- --- --- --- --- --- --- --- --- ... ... ... ... ... ... ... ... ... ... ... ... ...**

**94-567/G ... ... ... ... --- --- --- --- --- --- --- --- --- --- ... ... ... ... ... ... ... ... ... ... ... ... ...**

**94-567/H ... ... ... ... --- --- --- --- --- --- --- --- --- --- ... ... ... ... ... ... ... ... ... ... ... ... ...**

**94-567/I ... ... ... ... --- --- --- --- --- --- --- --- --- --- ... ... ... ... ... ... ... ... ... ... ... ... ...**

**94-567/J ... ... ... ... --- --- --- --- --- --- --- --- --- --- ... ... ... ... ... ... ... ... ... ... ... ... ...**

**94-567/K ... ... ... ... --- --- --- --- --- --- --- --- --- --- ... ... ... ... ... ... ... ... ... ... ... ... ...**

**94-567/L ... ... ... ... --- --- --- --- --- --- --- --- --- --- ... ... ... ... ... ... ... ... ... ... ... ... ...**

**91-1307/A-L AAC AGT --- --- --- --- --- --- --- --- --- --- --- --- ACC ATC TGG GGC CAC GGA ACC CTG GTC ACC GTC TCC TAC**

**93-2181/A-L CTG CGA CCT GTT CAC AGT GCC TCA GGG ACT TAT TAT GTT ATG GAC GTC TGG GGC AAA GGG ACC TCG GAC ACC GTC TCC TCA**
