# Supplementary material for: Grade I, II and III Follicular Lymphomas Express Ig VH Genes with Different Patterns of Somatic Mutation
Source: Pathol Oncol Res. 2020 Jul 23;26(4):2765–72. doi: 10.1007/s12253-020-00843-x (PMC7471144; doi:10.1007/s12253-020-00843-x)
Supplement: Supplementary file 2 — (DOC 26 kb) [file 12253_2020_843_MOESM2_ESM.doc]

**__CDR1_ _______CDR2______ ______CDR3______**

**1 10 20 30 40 50 60 70 80 90 100 110**

**. . . . . . . . . . . .**

**IGHV5-51 EVQLVQSGAEVKKPGESLKISCKGSGYSFT SYWIG-- WVRQMPGKGLEWMG IIYPGDSDTRYSPSFQG QVTISADKSISTAYLQWSSLKASDTAMYYCAR**

**87-784(GL) . .....-- .............. ................. ................................**

**87-784/A-C .............................. .....-- .............. ................. ................................ LENYGHH-------NY WGQGTLVTVSS**

**87-784/D-L .............................. N...A-- .............. M...H............ .........LT..................... .......-------.. ...........**

**IGHV3-48 EVQLVESGGGLVQPGGSLRLSCAASGFTFS SYSMN-- WVRQAPGKGLEWVS YISSSSSTIYYADSVKG RFTISRDNAKNSLYLQMNSLRAEDTAVYYCAR**

**94-567(GL) . .....-- .............. ................. ................................**

**94-567/A-C .............................. DFTL.-- ............I. N.....T.TN....... ...V....V....................... NSSA----------DY WGNGTLVTVSS**

**94-567/D .............................. DFTL.-- ............I. N...T.T.TN....... ...V....V....................... ....----------.. ...........**

**94-567/E .............................. DFTL.-- ............I. N.....T.TN....... ...V....V....................... ....----------.. .D.........**

**94-567/F .............................. DFTL.-- ............I. S.....T.TN....... ...V....V....................... ....----------.. ...........**

**94-567/G .............................. DF.L.-- ............I. N..-..NITD....... .....................V.......... ....----------.. ...........**

**94-567/H .............................. DF.L.-- ............I. N.T-..NITD....... .....................V.......... ....----------.. ...........**

**94-567/I .............................. DF.L.-- ............I. N..-..NITD.V..... .....................V.......... ....----------.. ...........**

**94-567/J ...........................I.. DF.L.-- ............I. N..-..NITD....... .....................V.......... ....----------.. ...........**

**94-567/K ...........................I.. DF.L.-- ............I. N..-..NITD....... .....................V.......... ....----------.. ...........**

**94-567/L ...........................I.. DF.L.-- C...........I. NM.-..NITD....... ........G............V.......... ....----------.. ...........**

**IGHV3-30 QVQLVESGGGVVQPGRSLRLSCAASGFTFS SYAMH-- WVRQAPGKGLEWVA VISYDGSNKYYADSVKG RFTISRDNSKNTLYLQMNSLRAEDTAVYYCAR**

**91-1307(GL) . .....-- .............. ................. ................................**

**91-1307/A-L .............................. R.T.F-- .............. L.....K.EH....... .C.......RD.....L....LN........T NS------------TI WGHGTLVTVSY**

**IGHV4-39 QLQLQESGPGLVKPSETLSLTCTVSGGSIS SSSYYWG WIRQPPGKGLEWIG SIY-YSGSTYYNPSLKS RVTISVDTSKNQFSLKLSSVTAADTAVYYCAR**

**93-2181(GL) . ....... .............. ...-............. ................................**

**93-2181/A-L .............................. .NIH... ....-......... TK.-...I.......M. .. V.A...N.R...S.T.L........F..S LRPVHSASGTYYVMDV WGKGTSDTVSS**
